# Centralization of ulna with wrist fusion for failure of reconstructed distal radius by allograft bone or prosthesis: A case report and literature review

**DOI:** 10.1097/MD.0000000000028272

**Published:** 2021-12-23

**Authors:** Zuchang Li, Yong Yang, Bin Li, Feng Li, Xingjian Huang

**Affiliations:** Hand Surgery Department, Beijing Jishuitan Hospital, Beijing, China.

**Keywords:** distal radius, failure, reconstruction, ulna centralization, wrist fusion

## Abstract

**Rationale:**

Centralization of the ulna is commonly used in the treatment of radius developmental deformity in children. The secondary distal radius deficiency in adults is different from the developmental deformity of the radius in children. There is no report on the ulna centralization with wrist fusion for the failure of the reconstructed distal radius by allograft bone or prosthesis for osteosarcoma in adults.

**Patient concerns:**

2 patients with a bone tumor on the distal radius underwent tumor resection and distal radius reconstruction by allograft bone or prosthesis and suffered distal radius collapsed fracture and radiocarpal joint dislocation accompanied with moderate pain, severe deformity, and poor grip and pinching power several months follow primary surgery.

**Diagnoses:**

X-ray images revealed collapsed fracture of distal radius and dislocation of the radiocarpal joint.

**Interventions:**

The 2 patients were operated on by the same technique under brachial plexus anesthesia. The allograft bone or prosthesis and the lunate were removed. The capitate was trimmed with a groove, and the cartilage surface with the subchondral bone of the distal ulna was resected to match the groove of the capitate. A straight plate with screws was used for internal fixation.

**Outcomes:**

Bone healing was achieved at 3 to 4 months after the surgery. After a minimum of 1-year follow-up, pain symptom was relieved, palmar flexion deformity was corrected, and grip and pinch strength were restored. The 2 patients were both satisfied with the improvement in appearance and function.

**Lessons:**

For adult patients who have failed resection and reconstruction of giant cell tumors, osteosarcoma, and other tumors of bone on the distal radius, ulna centralization is a simple and effective option.

## 1 Introduction

The forearm consists of radius and ulna, constituting the elbow with humerus proximally and the wrist with carpale distally. It has the function of force transmission and joint movement. The distal radius plays a more important role in the wrist than the distal ulna, however, the distal radius may be deficient due to trauma, infection, tumor, or prior surgery, which can deprive the wrist of much of its function, especially force transmission, and further affect the hand function. The most common cause is the giant cell tumor of the distal radius, an aggressive and potentially malignant lesion. Intralesional curettage with bone grafts, autologous bone or allogeneic bone graft, bone graft substitutes and/or polymethylmethacrylate, and artificial prosthesis implantation are common treatment methods.^[1–4]^ But some patients may have failed reconstructive surgery, complicated by radial carpal deformity with deficiency of distal radius, which seriously affects wrist and hand function. The next treatment troubles the surgeon.

Centralization of the ulna is mainly used in the treatment of radius developmental deformities in children (radial club hand deformity), an operation that persists until today in modified form, was performed by Sayre^[5]^ in 1894. Due to the abnormal development of the radius of the forearm, the radial deviation of the wrist gradually increases with growth, which seriously affects the function of the wrist and hand. Through the centralization of the ulna and the migration of the tendons, the radial deviation can be corrected, so that the hand can be stabilized on the forearm axis, continue to grow, and retain the function of the wrist, especially in severe deformities.^[6]^

The secondary distal radius deficiency in adults is different from the developmental deformities of the radius in children. The bone and soft tissue of adults have been fully developed and may be accompanied by the loss of soft tissues such as tendons. Therefore, there is no need to consider subsequent growth in treatment, especially when the first surgery failed. After that, re-treatment should strive to effectively improve function and better help patients return to daily life. Therefore, we applied centralization of the ulna with wrist fusion for the failure of the reconstructed distal radius to strive for more functional recovery for patients. The authors report 2 cases and review relevant literature to introduce this technique.

## 2 Case report

### 2.1 Case 1

**Figure 1:**
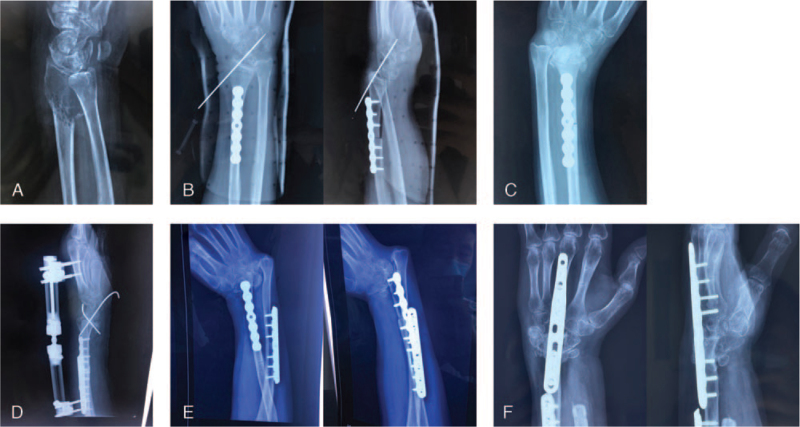
X-ray images of Case 1. A. Giant cell tumor of the left distal radius. B. Allogeneic bone transplantation. C. 5 months later, fractures and collapses of the distal radius and dislocation of the radiocarpal joint. D. 6 months after the first surgery, another surgery was performed. E. 22 months after the first surgery. F. Last follow-up, 15 months after ulna centralization.

**Figure 2:**
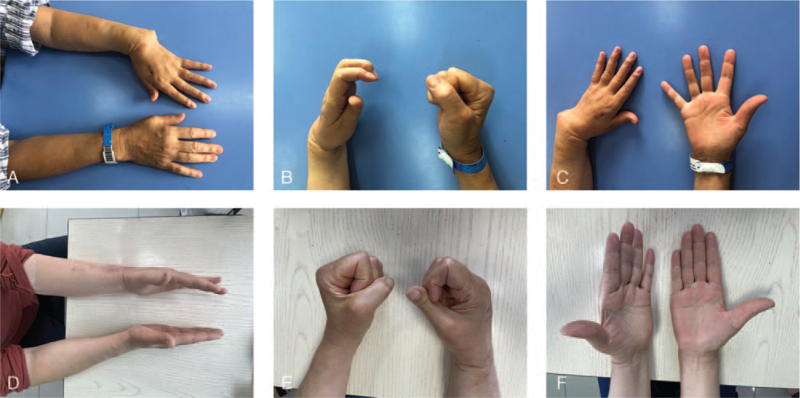
Forearm and hand deformity of Case 1. A, B, C. Hand function before ulna centralization. The patient cannot make a fist. D, E, F. 15 months after ulna centralization.

A 58-year-old female, 22 months after resection of giant cell tumor of the left distal radius and allogeneic bone transplantation. 22 months ago, due to a giant cell tumor of the distal radius on the left side (Fig. 1A), the patient underwent giant cell tumor resection and allogeneic bone transplantation (Fig. 1B) in the department of oncology. 5 months later, fractures and collapses of the distal radius and dislocation of the radiocarpal joint occurred (Fig. 1C); 6 months after the operation, another operation was performed with the ulna shortened, the radiocarpal joint reduced, and the external fixator and Kirschner wires were fixed (Fig. 1D); 22 months after the operation, further fracture collapsed and radio-carpal joint dislocated again, which made her to our department. The patient presented with severe deformity, accompanied by moderate pain, and extremely poor grip and pinching power of the affected limb (Fig. 2A, 2B, 2C).

X-rays showed that the distal radius was absorbed and collapsed, the palm of the wrist was dislocated to the radial side, and the distal ulna was protruding, close to the skin (Fig. 1E).

The patient was treated with surgery under brachial plexus anesthesia. During the surgery, the allograft bone and the lunate were removed and the capitate was trimmed with a groove, the cartilage surface and subchondral bone of the distal ulna were resected, matching with the groove of the capitate, and fixed by a straight plate and screws (Wrist fusion plate- straight, RFP-STR, TriMed, Inc) (Fig. 1F).

The follow-up was 15 months. Bone healing was achieved at 4 months after surgery, and the wrist dorsal extension angle was fixed 10°. At the last follow-up, the patient's pain symptoms were basically relieved, and the grip and pinch strength of the affected limb were significantly restored (Fig. 2D, 2E, 2F). The patient was satisfied with the improvement in appearance and function (Tables 1 and 2).

**Table 1:** Grip strength and pinch strength of patients.

	Grip strength (kg)			Pinch strength (kg)		
	Affected side			Affected side		
	Preoperative	Postoperative	Healthy side	Preoperative	Postoperative	Healthy side
Case 1	1.7	7.9	27.4	0	2.1	4.3
Case 2	8.4	22.5	36.2	1.8	4.5	5.6

**Table 2:** Wrist and forearm range of motion of patients.

	Wrist				Forearm			
	Flexion		Extension		Pronation		Supination	
	Preoperative	Postoperative	Preoperative	Postoperative	Preoperative	Postoperative	Preoperative	Postoperative
Case 1	70	0	-50	10	80	0	-60	0
Case 2	70	0	-60	15	40	0	-10	0

Informed consent was obtained from the patient for the purpose of publication.

### 2.2 Case 2

**Figure 3:**
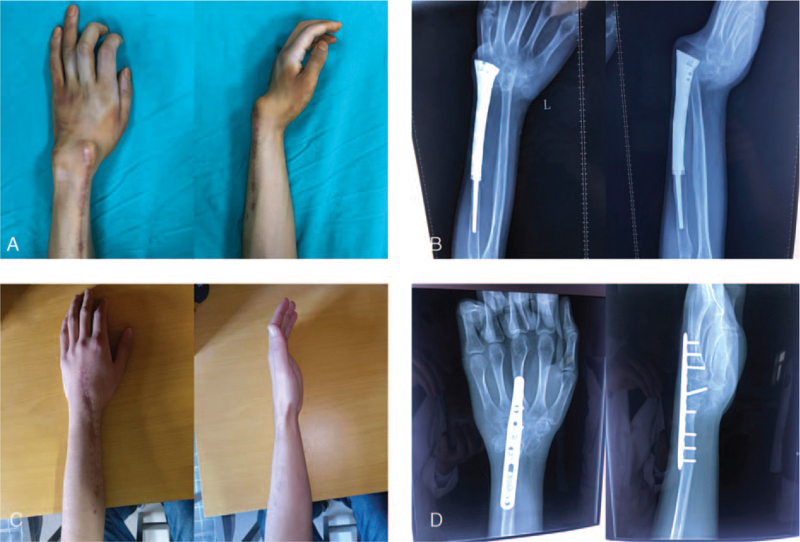
The affected side of Case 2. A, B. Before ulna centralization. C, D. 13 months after ulna centralization.

An 18-year-old male, 6 months after resection of the left distal radius osteosarcoma and implantation of the artificial prosthesis. 6 months ago, due to osteosarcoma of the left distal radius, the patient underwent osteosarcoma resection and three-dimensional printed prosthesis implantation in the department of oncology. Radio-carpal joint dislocation began 3 months later, and life was seriously affected 6 months after surgery. The patient had severe radial palmar flexion deformity, accompanied by mild pain, and extremely poor grip and pinching power (Fig. 3A).

X-rays showed that the radiocarpal joint was completely dislocated, the distal end shifted to the radio-volar side. The joints had completely lost their involute relationship (Fig. 3B).

The operation was performed in the same way, and the follow-up was 13 months. Bone healing was achieved 3 months after the operation, and the wrist dorsal extension angle was fixed 10°. At the last follow-up, there was no discomfort in the affected limb, and the grip and pinch strength were significantly restored (Fig. 3C, 3D). The patient was also satisfied with the improvement in appearance and function (Tables 1 and 2).

Informed consent was obtained from the patient as well for the purpose of publication. This study was approved by our institutional ethics board (Beijing Jishuitan Hospital Institutional Review Board

## 3 Discussion and literature review

There are few articles about the use of ulna centralization to treat adult distal radius defects. In 1996, Mukherjee et al^[7]^ reported 3 cases, of which 2 cases were recurrence treatment of giant cell tumor of bone after excision of fibula transplantation, and 1 case was treated with ulnar centralization directly after resection of giant cell tumor of bone. In 2008, Bhargava et al^[8]^ reported 1 case for the treatment of recurrence of giant cell tumor of bone after excision of fibula transplantation. In 2015, Ververidis et al^[9]^ reported 1 case for the treatment of recurrence of giant cell tumor of bone after curettage. The most reported cases were Bhagat et al^[10]^ and Meena et al^[11]^ In 2008, Bhagat et al^[10]^ reported 25 cases, of which 15 were recurrences of giant cell tumor of bone after excision and bone transplantation, and 10 were Enneking stage III giant cell tumors of bone. Due to the wide range of resections, bone grafting could not be performed and the ulna can be directly centralized. Among them, 2 patients needed additional bone grafts, and 6 patients were due to the prominent hardware and underwent the removal of the dorsal plate. The wrists of all patients were stable and the tumor did not recur. Compared with the healthy side, the grip strength of the affected side recovered to 70%. In 2016, Meena et al^[11]^ reported 10 cases of patients who had recurrence after curettage or resection and bone grafting of giant cell tumor of bone. All patients were treated with ulnar centralization for the second operation. The average follow-up time was 45 months, the average grip strength recovered to 45% of the healthy side, and the average bone healing time was 6 months. Therefore, according to previous literature reports, the operation of ulna centralization was mainly used in adults with giant cell tumors of the distal radius in patients who have failed the first wrist joint reconstruction. Through the fusion of the ulnar wrist joints, a stable wrist relationship can be reconstructed. In addition, we also applied this procedure to patients who failed after osteosarcoma resection and wrist reconstruction and obtained definite curative effects. Due to the short follow-up time and poor preoperative soft tissue conditions, the postoperative grip strength recovery did not reach the average value in previous reports, but it was significantly improved compared to the preoperative grip strength.

The most similar surgery to ulna centralization is ulna transposition. To preserve the rotation function of the forearm, the distal ulna is transplanted to the proximal radius stump based on centralization. In 2020, Chobpenthai et al^[12]^ conducted a systematic review of this surgery. The article included 12 studies, with an average follow-up time of 9 to 46.8 months, and the postoperative grip strength recovered to 38.9% to 86% of the healthy side. The pronation angle of the forearm was 70°-90°, and the supination angle was 55° to 90°. The main complications of surgery were delayed union and nonunion, with a total incidence of 16%. In addition, 4.44% of patients had an infection at the surgical site. The common point of ulna centralization and ulnar transposition is the ulna-carpal fusion. The fusion can provide good stability, effectively reduce postoperative pain, and obtain good hand function recovery. Although fusion can cause the loss of wrist flexion and extension, for patients who have removed most of the muscles and tendons due to tumor invasion or have obvious deformity and scar tissue of the surrounding muscles and tendons during the second operation, the fusion will obtain significant benefits. The difference between the two surgeries is that the centralization is less difficult and less traumatic, and it is easier to obtain the stability of the forearm and reduce the occurrence of complications; while the transposition increases the fusion site of the bone graft, and there is a risk of delayed or nonunion of the corresponding site, but it retains the pronation and supination function of the forearm for the patient. In the clinic, the patient's condition, age, physical condition, occupation, demand for forearm function, economic conditions, and other comprehensive considerations can be used to select the surgical method.

## 4 Conclusion

Ulna centralization has such advantages: low surgical difficulty, good stability, significant improvement in wrist deformities, improved hand function, and avoidance of donor site damage. But it also has disadvantages: loss of wrist flexion and extension function after fixation and fusion and loss of forearm rotation function. For adult patients who have failed resection and reconstruction of giant cell tumors, osteosarcoma, and other tumors of bone on the distal radius, ulna centralization is a simple and effective option.

## Author contributions

**Conceptualization:** Yong Yang.

**Data curation:** Bin Li, Feng Li, Xingjian Huang.

**Formal analysis:** Zuchang Li.

**Investigation:** Yong Yang, Bin Li, Feng Li, Xingjian Huang.

**Methodology:** Yong Yang.

**Project administration:** Yong Yang.

**Supervision:** Yong Yang.

**Visualization:** Zuchang Li, Bin Li, Feng Li, Xingjian Huang.

**Writing – original draft:** Zuchang Li.

**Writing – review & editing:** Yong Yang.
